# Influence of Steel Plates and Studs on Shrinkage Behavior and Cracking Potential of High-Performance Concrete

**DOI:** 10.3390/ma12030342

**Published:** 2019-01-22

**Authors:** Lepeng Huang, Jianmin Hua, Ming Kang, Qiming Luo, Fengbin Zhou

**Affiliations:** 1 School of Civil Engineering, Chongqing University, Chongqing 400045, China; huang_lepeng@cqu.edu.cn (L.H.); kmingcqu@cqu.edu.cn (M.K.); luoqiming@cqu.edu.cn (Q.L.); 20141613158@cqu.edu.cn (F.Z.); 2 Key Laboratory of New Technology for Construction of Cities in Mountain Area (Chongqing University), Ministry of Education, Chongqing 400045, China

**Keywords:** high-performance concrete shrinkage, steel plate, stud, cracking potential, steel-plate reinforced concrete shear wall

## Abstract

To help designers develop solutions to overcome the cracking problem in steel-plate-reinforced concrete composite shear walls due to the concrete shrinkage, the influence of steel plates and studs on the shrinkage behavior of high-performance concrete (HPC), including restrained shrinkage strain, shrinkage strain gradient, and cracking potential, were theoretically and experimentally investigated in this study. A model for theoretical analysis was used to research the shrinkage performance of concrete that was restrained by steel plates and studs. The major parameters involved in the experiments include the thickness and material elastic modulus of the steel plate, in addition to the diameter, height, and number of studs. It was found that the shrinkage of HPC decreases and its potential cracking increases with the increase of thickness and elastic modulus of the steel plate, and the diameter, height, and number of studs. The restraining effect of the steel plate and stud on the HPC shrinkage decreases with the distance of their respective locations. It demonstrates that the HPC near a steel plate and stud is prone to crack compared with that far away from the steel plate and stud. This potential could be reduced by uniformly restraining the HPC.

## 1. Introduction

The volume of concrete can decrease, even in the absence of external load, with the reduction in the internal humidity of concrete caused by hydration and drying effects. Such reduction in the concrete volume is called shrinkage. If the shrinkage is restrained using a restraint, reinforcement, or a combination of steel plate and studs, a restraining tensile stress is generated in the concrete. When the tensile strain of concrete exceeds the ultimate tensile strain, concrete cracking occurs [1].

In recent years, steel-plate-reinforced concrete composite shear walls (SPRW) have been widely used for super high-rise buildings, such as PINGAN International Finance Centre (592.5 m) and Shanghai Tower (632 m) in China, because of their good structural performance [2]. Compared to the conventional reinforced concrete shear wall, SPRW consist of high-performance concrete (HPC), embedded steel plates, and densely distributed studs. The shrinkage in HPC, especially in the early age of the concrete, is much higher than that in normal concrete due to the low water/cement ratio of HPC [3,4,5]. Moreover, the concrete used in the SPRW is restrained by reinforcement and a combination of steel plate and studs, rather than only reinforcement as in traditional reinforced concrete shear walls. Higher free shrinkage and a complex restraining effect thus lead to a higher cracking risk in SPRW.

To prevent concrete cracking due to shrinkage, a basic requirement is to study the influence of the restraint on the concrete shrinkage. Several studies have attempted to investigate the influence of reinforcement on concrete shrinkage behavior, and many useful results have been obtained [6,7,8,9,10,11,12,13,14,15,16]. As revealed in the results of test performed by Gao et al. [6], the restraining effect made by the reinforcement bar on the concrete shrinkage is enhanced with a rise in the reinforcement ratio. Yoo et al. [7] revealed that the reductions in the autogenous shrinkage stress, the degree of restraint, and the cracking potential of concrete could be made possible through the use of reinforcing bars with lower stiffness. Huang et al. [8] carried out a study that was aimed at figuring out the influence of reinforcement configuration on the shrinkage and cracking potential of high-performance concrete. The results from this study indicated that the restraining effect of the reinforcement on concrete shrinkage declined as the distance to the reinforcing bar increased. However, the influence of steel plates and studs on the concrete shrinkage and cracking potential has not been extensively analyzed. Although Nie et al. [9] made a report that discovered that, due to the restraining effect of steel plate, the concrete shrinkage tends to exert a greater influence on the cracking load and cracks width of composite structures than traditional reinforced concrete structure, a lack of detailed discussion remains regarding the restraining effect of steel plates. Zeng [10] undertook an analysis of the restraining tensile stress induced in SPRW using numerical simulation, which suggested that studs were acting in a way that caused the restraining tensile stress to surge in the SPRW, with the highest cracking potential of concrete being pinpointed as being near the studs. However, there was no clear conclusion drawn as to the theory for explaining these results. Hryniuk et al. [11] investigated the influence of lateral restraint on structural response of steel-concrete (SC)composite wall numerically. They revealed that the lateral restraint effects stemming from differential expansions of the steel faceplates and the concrete cores comprising SC elements play an important role in the structural response of steel-concrete composite wall to the loads. Although the restraining effect of the steel plate and stud is not completely unexpected, the restraining mechanism of steel plate and stud to concrete shrinkage and precise contributing role of steel plate and studs in the level of risk of concrete cracking is still unclear. Until now, owing to the lack of related research, the clauses for crack control for SPRW in the Chinese structural codes have been based on results of research on reinforcements [17,18,19]. Although these clauses have proven to work in some cases, they are not applicable in several others. Consequently, large-area cracking occurred in many SPRW during the construction period [20].

Thus, this study aims to investigate the influence of steel plates and studs on shrinkage behavior and cracking potential of high-performance concrete to help designers develop solutions to overcome the cracking problem in SPRW. To highlight the effect of steel plates and studs on the shrinkage strain, shrinkage gradients, and cracking potentials of the HPC, this study starts with suggesting a model of theoretical analysis to establish the actual shrinkage performance of concrete restrained by steel plates and studs. Subsequently, the relevant experiments are conducted as a way to validate the model and further study the shrinkage performance, as well as the cracking potential of concrete restrained using steel plates and studs. The major parameters involved in these experiments include the thickness and material elastic modulus of the steel plate, in addition to the diameter, height, and number of studs. An evaluation is carried out of the strain at multiple positions on the specimen cross-sections, different internal relative humidities (RHs) and internal temperatures, and the changes in HPC material properties.

## 2. Analytical Model

**Figure 1:**
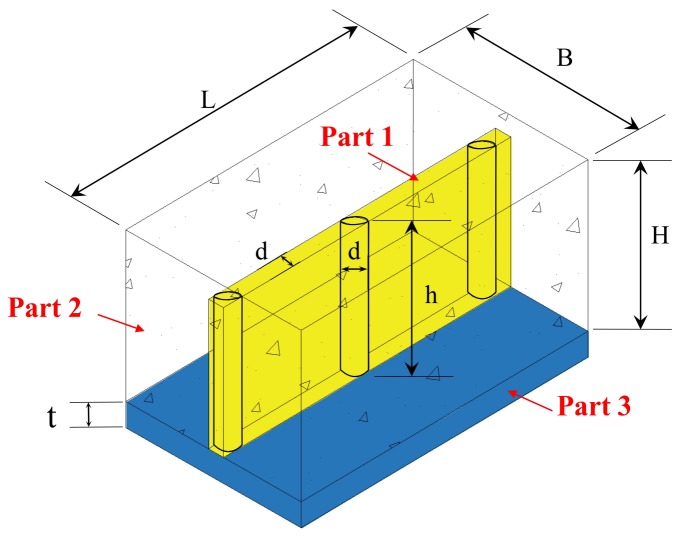
Model.

An analysis model was proposed to analyze the shrinkage performance of concrete restrained by a steel plate and stud with reference in previous study [12], as shown in Figure 1. The length, thickness, and width of the steel plate were *L*, *t*, and *B*, respectively. The nominal diameter of the stud was *d*, and the height was *h*. The *n* denotes the number of studs (Figure 1 shows three studs). To simplify the analysis, the nominal diameter was used to replace the head diameter of the stud. The height of the concrete in the model was *H*. The total length, height, and width of the model were thus *L*, *H + t*, and *B*, respectively. It was assumed that there was no relative sliding between the concrete, steel plate, and studs.

This model focused on shrinkage in the length direction. Thus, the stress in the width and height direction, and their influence on the shrinkage strain and stress in the length direction were not considered in the modelling procedure. Since the stud represents a discontinuity in the shrinkage direction of the concrete, it is difficult to directly analyze the restraining effect of the studs. Thus, the model was divided into three parts: Part 1 included the stud and the concrete in the range of the stud diameter and height, which is indicated by yellow region in Figure 1. Part 2 included the concrete that was beyond the range of the stud diameter and height. Part 3 included the steel plate (blue region in Figure 1). The head of the stud was ignored in this model. Since the stud head only occupied a small proportion of the entire stud, and the difference of radius between the screw and the head was relatively small, the error caused by the removal of the stud head was acceptable.

When concrete shrinkage occurred in Part 2, Equation (1) can be written as follows, according to the requirement for force balance:

(1) $$E_{2}(\varepsilon_{\mathrm{sh}}-\varepsilon_{r})A_{2}=E_{3}\varepsilon_{r}A_{3}+E_{1}\varepsilon_{r}A_{1}$$

where ε_sh_ is the free shrinkage strain of concrete. ε_r_ is the measure strain in the restrained specimens. *E*_1_ is the elastic modulus of Part 1. *E*_2_ is the elastic modulus of Part 2 (herein, it equals to the elastic modulus of concrete (initial tangent modulus) *E*_c_). *E*_3_ is the elastic modulus of Part 3 (herein, it equals to the elastic modulus of steel plate (initial tangent modulus), *E*_st_). *A*_1_, *A*_2_, and *A*_3_ denote the cross-sectional area of Parts 1, 2, and 3, respectively.

Accordingly, the strain in the restrained specimens can be described using:

(2) $$\varepsilon_{r}=\frac{\varepsilon_{\mathrm{sh}}}{\frac{E_{3}}{E_{2}}•\frac{A_{3}}{A_{2}}+\frac{E_{1}}{E_{2}}•\frac{A_{1}}{A_{2}}+1}=\frac{\varepsilon_{\mathrm{sh}}}{\frac{E_{\mathrm{st}}}{E_{c}}•\frac{A_{3}}{A_{2}}+\frac{E_{1}}{E_{c}}•\frac{A_{1}}{A_{2}}+1}$$

**Figure 2:**
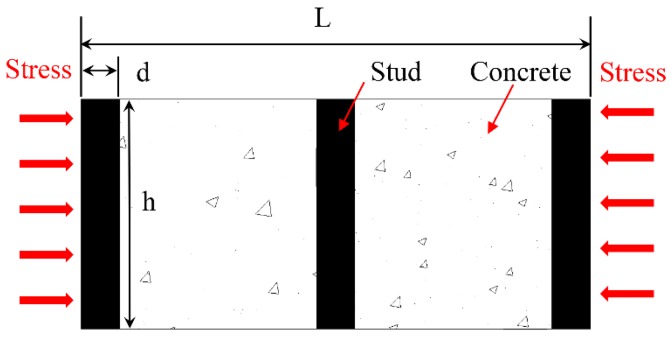
Model for the analysis of Part 1.

One needs to determine *E*_1_ to obtain the strain in the restrained specimens. A separate analysis considering only Part 1 was performed, as shown in Figure 2. The projected area was used to represent the cross-sectional area of the stud. 

Given that stress *σ* acts on Part 1, the total deformation can be described using:

(3) $$\Delta L=\varepsilon_{t}nd+\varepsilon_{c}(L-nd)$$

where Δ*L* is the total deformation of Part 1. ε_t_ and ε_c_ are the strain of the studs and the concrete, respectively.

ε_t_ and ε_c_ are expressed using:

(4) $$\left\{ \begin{array}{l} \varepsilon_{t}=\frac{\sigma }{E_{t}}\\ \varepsilon_{c}=\frac{\sigma }{E_{c}} \end{array}\right.$$

where *E*_t_ is the elastic modulus (initial tangent modulus) of the stud.

The strain in Part 1 (ε_t−c_) can therefore be written as:

(5) $$\varepsilon_{t-c}=\frac{\frac{\sigma }{E_{t}}nd+\frac{\sigma }{E_{c}}(L-nd)}{L}$$

According to the analysis of the restraining effect of reinforcement in a previous study [21], Part 1 can be regarded as a mixture. The average strain of the mixture (ε_mix_) under stress σ is then:

(6) $$\varepsilon_{\mathrm{mix}}=\frac{\sigma }{E_{1}}$$

According to Equations (4) and (5), Equation (6) is re-written as:

(7) $$\varepsilon_{\mathrm{mix}}{=\varepsilon }_{t-c}=\frac{\sigma }{E_{1}}=\frac{\frac{\sigma }{E_{t}}nd+\frac{\sigma }{E_{c}}(L-nd)}{L}$$

Substituting ρ’ = *nd*/*L* into Equation (7), *E*_1_ can be expressed as:

(8) $$E_{1}=\frac{E_{c}E_{t}}{\rho^{\prime }E_{c}+(1-\rho^{\prime })E_{t}}$$

By substituting Equation (8) into Equation (2), and on the basis of the unidirectional (length direction) analysis method used in this study, the strain of the restrained specimens can be described using:

(9) $$\varepsilon_{r}=\frac{\varepsilon_{\mathrm{sh}}}{\frac{E_{\mathrm{st}}}{E_{c}}•\frac{A_{3}}{A_{2}}+\frac{E_{t}}{\rho^{\prime }E_{c}+(1-\rho^{\prime })E_{t}}•\frac{A_{1}}{A_{2}}+1}$$

Equation (9) indicates that the strain of the concrete restrained by the steel plate and studs decreases with the increase in the area and material elasticity modulus of steel plate, and the diameter, height, number, and elasticity modulus of the studs, when the size of the specimens and the free shrinkage of the concrete are constant. Equation (10) can be used to analyze the shrinkage performance of concrete restrained only by a steel plate (without studs):

(10) $$\varepsilon_{r}=\frac{\varepsilon_{\mathrm{sh}}}{\frac{E_{\mathrm{st}}}{E_{c}}•\frac{A_{3}}{A_{2}}+1}$$

## 3. Experimental Setups

To verify the analytical model and obtain more information regarding the restraining effect of the steel plate and studs on the shrinkage behavior and cracking potential of HPC, experiments were conducted as listed in the subsequent sections.

### 3.1. Materials and Mix Proportions

The details of mix proportions are presented in Table 1. Portland cement and fly ash were used as cementitious materials. The chemical compositions and physical properties of the Portland cement and fly ash are listed in Table 2. Crushed limestone with a maximum nominal size of 20 mm was used as the coarse aggregate. The fineness modulus of the fine aggregate (quartz sand) was 3.0. The target 28-day cubic compressive strength of the concrete was 80 MPa.

### 3.2. Measurement of Shrinkage, Relative Humidity, and Temperature

**Figure 3:**
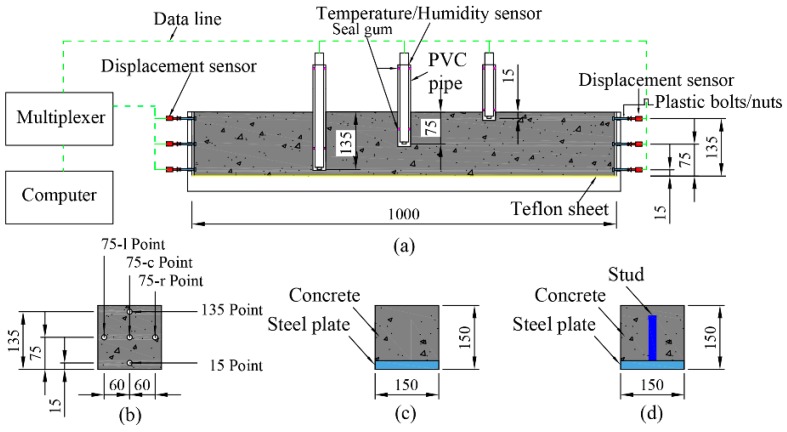
Shrinkage, temperature, and RH test setup for the HPC (mm): (**a**) schematic of the Plexiglas model, (**b**) distribution of the measurement position, (**c**) specimens with steel plate only, and (**d**) specimens with steel plate and stud.

Two types of specimen were developed: one restrained only by a steel plate, and the other restrained by a steel plate and studs. The specimens were made using a Plexiglass mold. The dimension the specimens was 150 mm × 150 mm × 1000 mm. After initial setting, the Plexiglas mold at the side face of the specimens were moved, and the bottom of all the specimens was covered with a 1-mm-thick Teflon sheet to ensure that the specimens were restrained only by the steel plate and studs (Figure 3). The specimen details are listed in Table 3 and the material properties of the steel plate and stud are listed in Table 4. In Table 4, “P” means the specimen was not restrained by stud and steel plate. “Sp” means the specimens were restrained by steel plate only. “St” means the specimens were restrained by steel plate and stud. In order to be consistent with the analytical model, in the experiments, the head of the studs were removed.

The precise information concerning the deformation gradient caused by the drying effect, especially in the restrained specimens, remains unclear [8]. To avoid the influence of the drying effect, all surfaces of specimens were covered by aluminum tape after initial setting to create a sealed curing condition.

The shrinkage of the specimens was measured at five different points, at three different heights (15, 75, and 135 mm from the bottom of the specimen). Three of these points were longitudinally located at 75 mm from the bottom. These are denoted, according to the measurement points, as “15 position,” “75-c position,” “75-l position,” “75 position-r,” and “135 position,” as shown in Figure 3. Linear variable differential transducers (LVDTs), with a measurement range and accuracy of 2 mm and 1 μm, respectively, were mounted on two longitudinal ends of each specimen to measure the external deformation of the specimens. To ensure the LVDTs could measure the deformation, some nuts were precast into the specimens. After the specimens were sealed using aluminum tape, plastic bolts were screwed into the nuts. In this way, the sensory bar of the LVDTs was directly in contact with the bolts.

The experiments were conducted under the following conditions: constant relative humidity (RH) of 60 ± 5% and temperature of 23 ± 1 °C. The RH and temperature of the specimens were measured according to a method suggested by Zhang et al. [22].

### 3.3. Measurement of Basic Properties

The cube compressive strength and elastic modulus of the concrete at ages of 3, 7, 14, 21, and 28 days were observed, and the results are presented in Table 5.

## 4. Results and Discussion

### 4.1. Free Shrinkage

**Figure 4:**
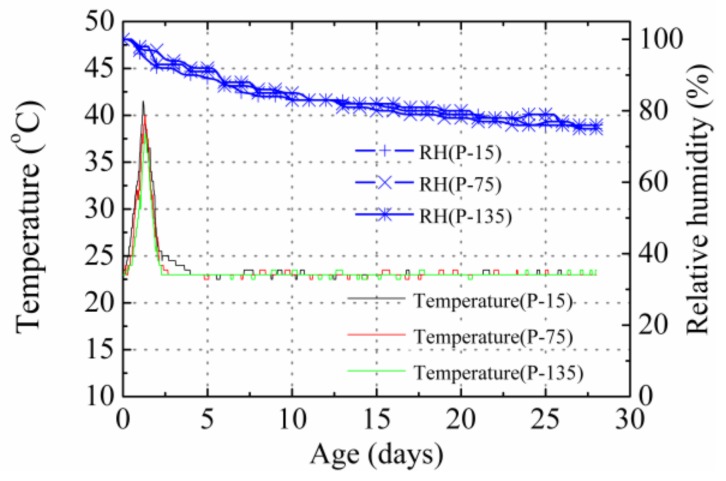
Temperature and RH in the P specimens.

Figure 4 shows the temperature and RH in the P specimens. Due to the intensive hydration effect, the internal temperature increased rapidly in the early age (i.e., before 20 h). At the age around 28.5 h, the temperatures at the 15, 75, and 135 positions successively reached their maximum values, at 41.5, 40.5, and 38 °C, respectively. Subsequently, the temperature decreased and attained equilibrium with the environment temperature at an age of approximately 60–70 h. Owing to the different distances of the thermal sensors shown in Figure 3 to the radiating surface, temperature gradients were recorded in the specimens.

**Figure 5:**
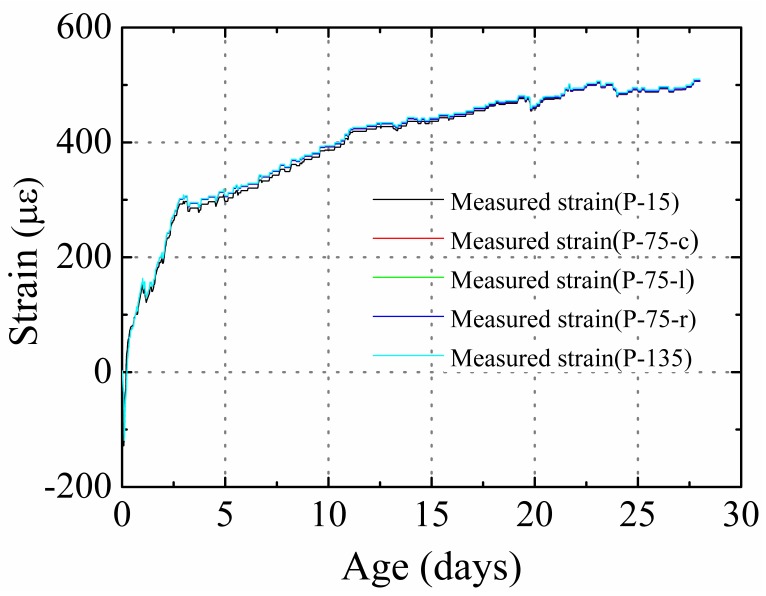
Measured autogenous shrinkage strain in the P specimens.

Figure 5 presents the measured autogenous shrinkage strain in the P specimens. The strain gradient caused by temperature gradient was eliminated according to the method suggested by Huang et al. [8]. As the specimens were sealed using aluminum tape after initial setting such that no moisture exchange occurred between the concrete and outside environment. In this situation, hydration was the only reason for the reduction in moisture content, and hydration was uniform throughout the specimen. As a result, no obvious shrinkage and RH gradient were found in the plain specimens, and the shrinkage strain and RH obtained at each position were nearly equivalent, as shown in Figure 4 and Figure 5. The total shrinkage of the specimens at the 15, 75-c, 75-l, 75-r, and 135 positions after 28 days was 506, 500, 512, 518, and 520 με, respectively.

### 4.2. Specimen Restrained Only by the Steel Plate

**Figure 6:**
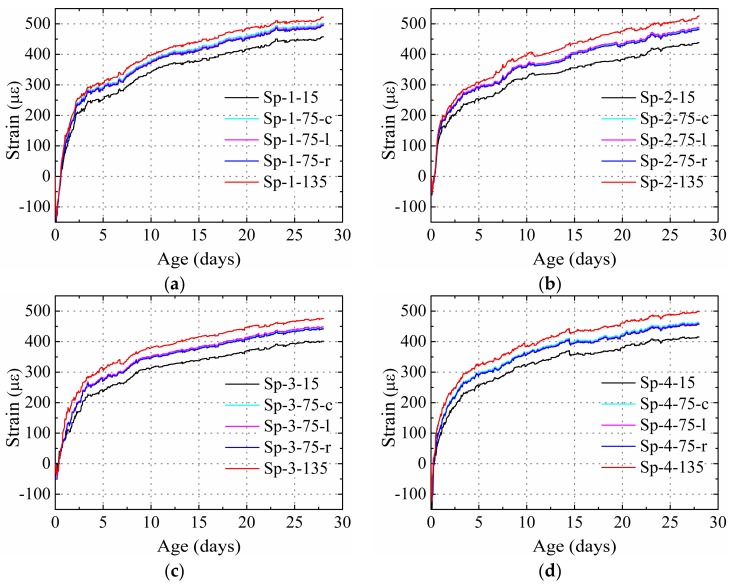
Measured strain for specimens restrained only by steel plate: (**a**) Sp-1, (**b**) Sp-2, (**c**) Sp-3, and (**d**) Sp-4.

Figure 6 presents the distribution of the strain of specimens restrained only using steel plates, with different thicknesses and materials. The strain gradient caused by the temperature gradient was eliminated. The test results highlight three characteristics of the shrinkage performance of the HPC restrained by steel plate, compared to that of the plain concrete: (1) For steel plates with the same material, the strain obtained at the same measurement position decreased with the increase of thickness in the steel plate. For example, at the age of 657 h, the shrinkages obtained at the 15 position for Sp-1 (4 mm), Sp-2 (6 mm), and Sp-3 (10 m) were 457, 438, and 400 με, respectively. (2) By comparing the strain obtained from Sp-3 and Sp-4, in which steel plates of the same thickness but different materials were used, it was found that the strain of the specimens decreased with the increase of elastic modulus in the material. For instance, at the age of 28 days, the shrinkages obtained at the 15, 75-c, 75-l, 75-r, and 135 positions for Sp-3 with the steel plate elastic modulus 20.6 × 10^4^ MPa were 401, 443, 447, 438, and 475 με, which were 23, 30, 18, 10, and 22 με lower, respectively, than the values obtained from Sp-4 with a steel plate elastic modulus of 15.4 × 10^4^ MPa. 

The width of the specimens was constant in the present study and the area of the steel plate was changed with the change in thickness. The test results are consistent with the theoretical derivation given by Equation (10): the strain of the HPC restrained by steel plates decreased with the increase in area and elastic modulus of material. A lower strain indicates higher restraining effect on the shrinkage of concrete. Thus, it can be concluded that the restraining effect of steel plate on the HPC shrinkage strain increased with the increase in area and material elastic modulus of the steel plate.

**Figure 7:**
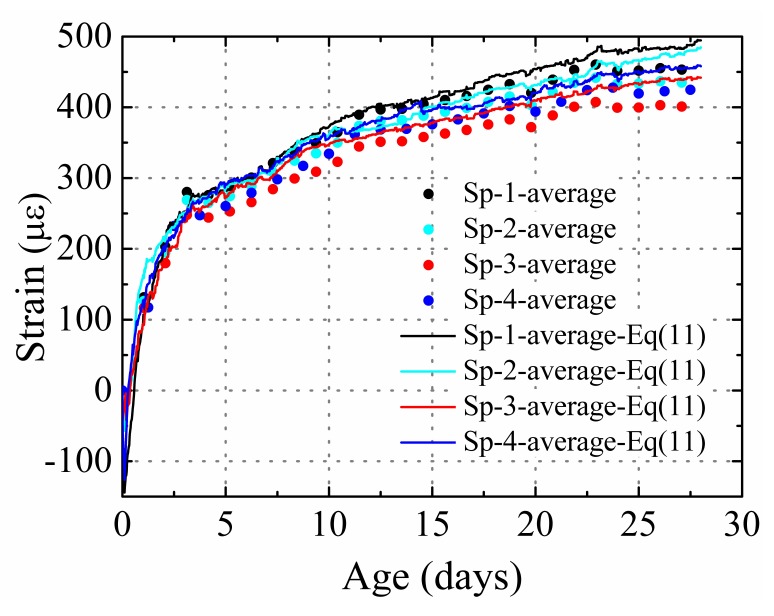
Comparison between the experimental results and those calculated using Equation (11).

Figure 7 shows a comparison of the average measured strains (average strain at the 15, 75-c, 75-l, 75-r, 75-c, and 135 positions) of the restrained specimens and the values calculated using Equation (10). The concrete elastic modulus at different ages are listed in Table 5 (the concrete elastic moduli at different ages were determined by interpolation). In Figure 7, it is observed that the calculation results agree well with the test results (Table 6). This indicates that Equation (10) can be used to calculate the average cross-section strain in HPC specimens restrained only by steel plates, and Equation (10) can be modified to be:

(11) $${\bar{\varepsilon }}_{r}=\frac{{\bar{\varepsilon }}_{\mathrm{sh}}}{\frac{E_{\mathrm{st}}}{E_{c}}•\frac{A_{3}}{A_{2}}+1}$$

where ${\bar{\varepsilon }}_{r}$ is the average strain of the cross section of the restrained specimens and ${\bar{\varepsilon }}_{\mathrm{sh}}$ denotes the average shrinkage of the plain concrete specimens.

The third characteristic is the presence of a deformation gradient even when the strain gradient caused by temperature was eliminated, and no moisture gradient was found due to the sealed curing condition, according to the results obtained from the plain specimens (Figure 5). A similar phenomenon has been observed by Wald et al. [14] while investigating the expansion behavior of reinforced concrete specimens. For all the specimens, the strain increased with the increase of the height of the specimens. The strain can reflect the restraining effect of the steel plate, which can be measured by the degree of restraint, as given by Equation (12) [8]:

(12) $$R=100\%\times (1-\frac{\varepsilon_{r}}{\varepsilon_{\mathrm{sh}}})$$

**Figure 8:**
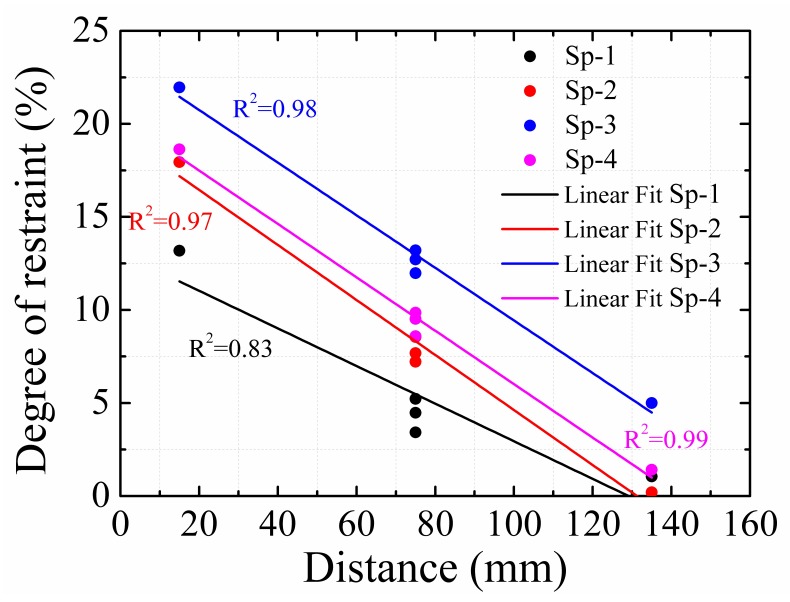
Distribution of average degree of restraint throughout the age of the specimen.

Figure 8 shows the relationship between the average degree of the restraint of each measurement position at different ages, and the distance from each measurement position to the bottom of the specimen. It was found that the degree of restraint of the steel plate decreased with the increase in distance. Similar trends were observed for those at positions of 75-c, 75-l and 75-r, which were equidistant to the bottom. The results indicate that the restraining effect of the steel plate on the HPC shrinkage decreased with the distance increase of HPC to the steel plate. 

**Figure 9:**
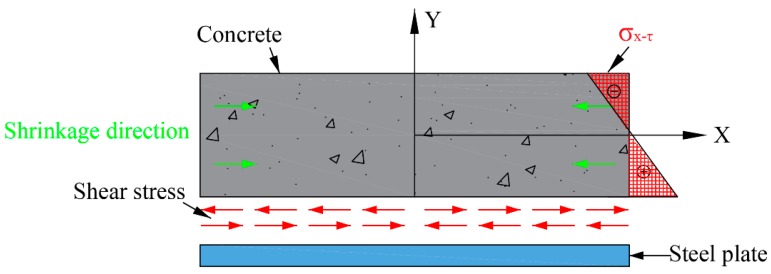
Analytical model of concrete restrained by a steel plate.

Figure 9 shows the analytical model for explaining how the degree of restraint of the steel plate decreased with the increase of distance from the concrete to the steel plate. For the restrained specimens, when concrete shrinkage takes place, shear stress is generated in the interface between the steel plate and concrete because of the bond effect. As a result, the shear stress results in the generation of normal stress in the x-direction in the specimens. In this study, only the shrinkage strain and stress development in length direction (*x*-direction) were considered. In this situation, the development law of the normal stress in the *x*-direction can be described using:

(13) $$\sigma_{x-\tau }=\frac{-24\tau xy}{H^{2}}+\frac{2\tau x}{H}$$

where σ*_x_*_-τ_ is the normal stress caused by the shear stress and τ is the shear stress that occurs at the interface of concrete and steel plate.

According to Equation (13), for a constant coordinate value of x, the normal stress changed with the y-coordinate. In the present study, at first, the normal stress had a direction opposite to that of the shrinkage of concrete, and the value of the normal stress decreased with increase of value in the y-coordinate. Then, the normal stress direction changed to be the same as that of the shrinkage, and the absolute value of the normal stress increased with the y-coordinate. In this condition, the strain in the restrained specimens increased with the increase of distance from the point to the bottom of the specimens. In other words, the restraining effect of the steel plate on the HPC shrinkage decreased with the increase in the distance to the steel plate. The shrinkage obtained at the 75-c, 75-l, and 75-r positions was similar as they were equidistant from the bottom of the specimen. 

As observed in Figure 8, the restraining effect of steel plates on HPC shrinkage was linearly related to the distance of HPC to the steel plate, and the change law of the normal stress proves the rationality of this relationship. However, due to the complexity of the capillary stress, shear stress, and the deformation law of the concrete under these stresses, the use of Equation (13) to calculate the decrease law of the restraining effect was difficult and inappropriate. According to the test results, Equation (14) was proposed to improve the accuracy in the calculation:

(14) $$R_{S}=R_{0}+mS$$

where *R*_s_ is the degree of restraint when the distance to the steel plate is *S*, *R*_0_ is the degree of restraint at the location of the steel plate, and *m* is the coefficient.

The average degree of restraint of the cross section of the steel plate to the HPC ($\bar{R}$) can be obtained using Equations (11) and (12). As the decrease in the restraining effect of the steel plate was linear, the average degree of restraint of the cross section can also be described using:

(15) $$\bar{R}=\frac{R_{0}+R_{H}}{2}$$

where *R*_H_ is the degree of restraint at the surface of the specimens.

The degree of restraint at the location of the steel plate can be calculated using:

(16) $$R_{0}=\frac{\varepsilon_{\mathrm{sh}}(2-mH)-2\bar{\varepsilon_{r}}}{2\varepsilon_{\mathrm{sh}}}$$

According to Equations (14)–(16), the shrinkage distribution in the specimens caused by the restraining effect decreases and is expressed using:

(17) $$\varepsilon_{r-s}=\varepsilon_{\mathrm{sh}}(1-R_{s})$$

where ε_r-s_ is the strain when the distance to the steel plate is *S*.

**Figure 10:**
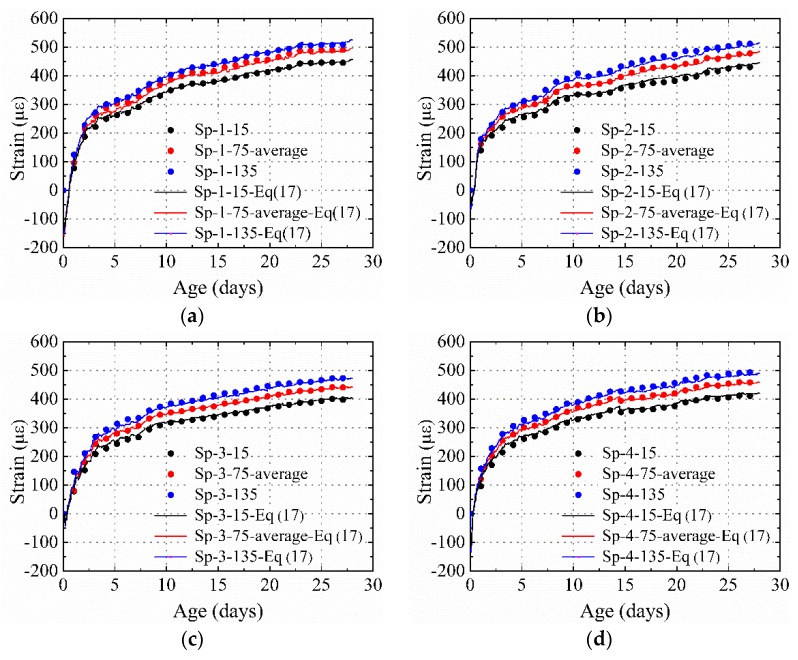
Comparison between results of experiment and those obtained using Equation (17): (**a**)Sp-1, (**b**) Sp-2, (**c**) Sp-3, and (**d**) Sp-4.

Figure 10 shows the comparison between the experimental results and those calculated using Equation (17). In the present study, *m* was equal to −0.001/mm. Because the 15 position was close to the center of the cross-section of the steel plate, the degree of restraint at the location of the steel plate was taken as the same value with that at the 15 position. It was found that Equation (17) was effective at predicting the shrinkage distribution at the cross section of specimens when the free shrinkage and the characteristics of the steel plate, such as area and elastic modulus, were determined. It should be noted that Equation (15) to Equation (17) could accurately predict shrinkage distribution when the steel plate had a strong restraining effect that restrains the entire cross section of specimens.

### 4.3. Restrained by Steel Plate and Stud

**Figure 11:**
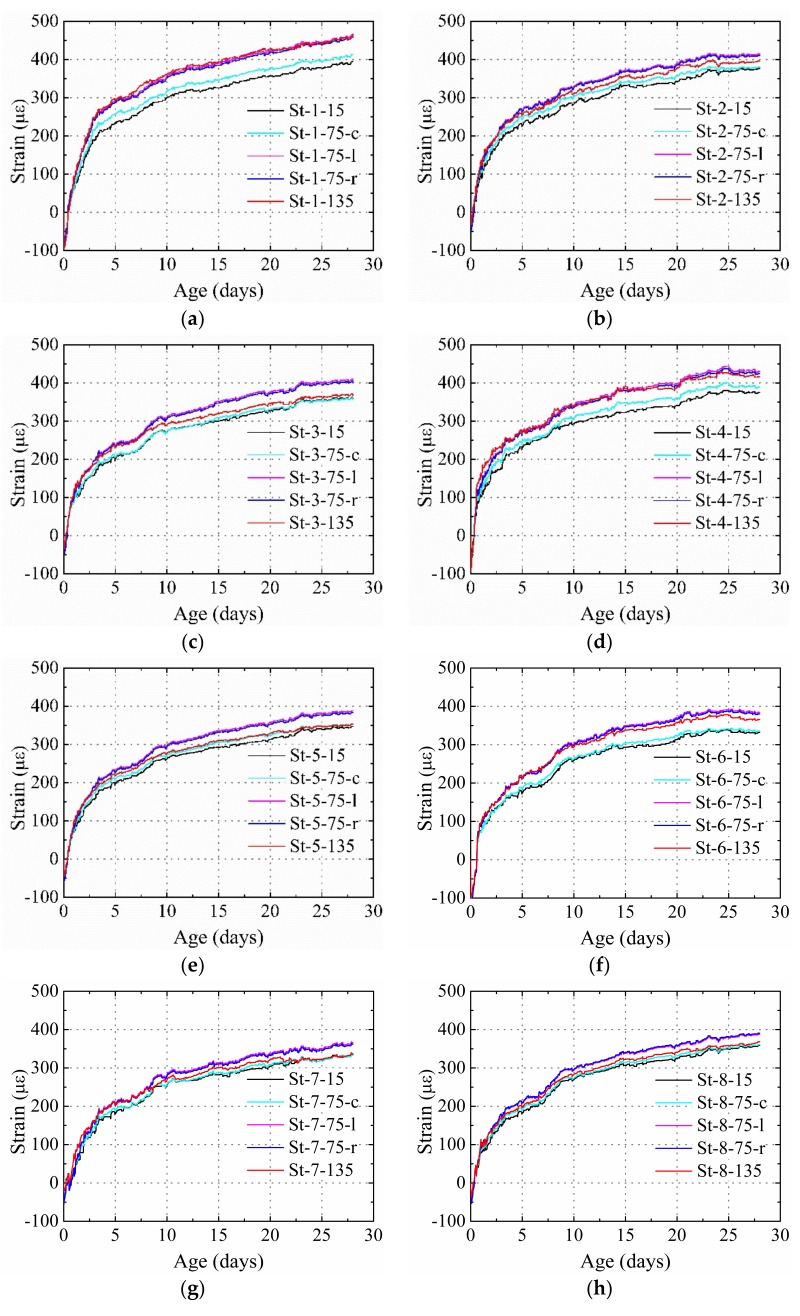
Measured strain for the specimens restrained by steel plate and stud: (**a**) St-1, (**b**) St-2, (**c**) St-3, (**d**) St-4, (**e**) St-5, (**f**) St-6, (**g**) St-7, and (**h**) St-8.

Figure 11 presents the strain distribution for the specimens restrained by the steel plate and the stud. The thickness of the steel plate was 10 mm for all specimens. The deformation caused by temperature was also eliminated. It was found that: (1) the shrinkage decreased with the increase of diameter of the stud when the number and height of the stud remained constant; (2) in specimens with the same height and diameter of stud, the shrinkage at the same position decreased with the increase of stud number, as shown in Figure 11b,c; (3) when the studs in the specimens had the same diameter and number but different heights, higher strains were found for each measurement position in specimens with a lower stud height. These test results present the same trends as the results of the theoretical derivation. The results indicate that an increase in the stud diameter, height, and number would increase the restraining effect of the stud on the HPC. 

**Figure 12:**
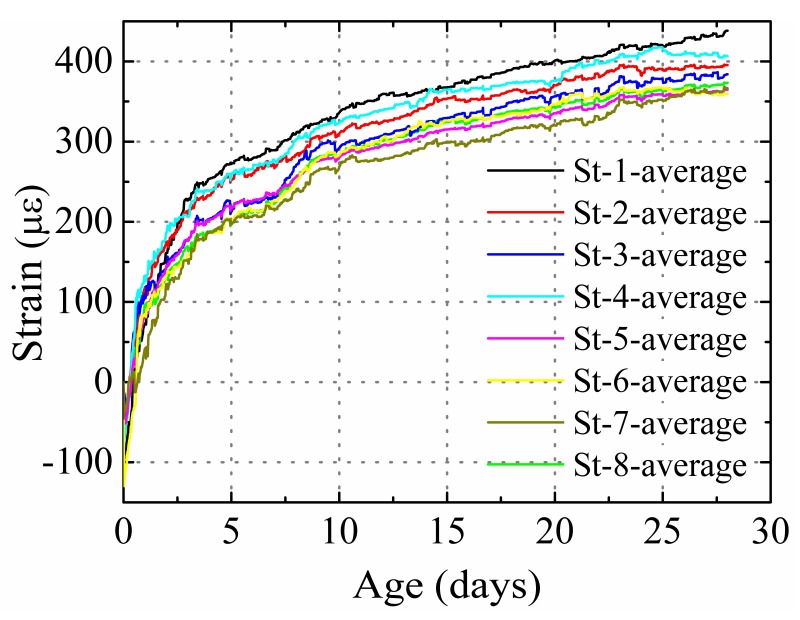
Average shrinkage of the specimens.

According to Equations (2) and (9), the change in the stud diameter, height, and number indeed affects the elastic modulus and cross-sectional area previously obtained in Part 1. A low strain is obtained when a higher product of *E*_1_ and *A*_1_ of the specimens was used. The product of *E*_1_ and *A*_1_ for St-1, St-4, St-2, St-3, St-6, St-5, and St-7 increased in order, and the average shrinkage of these specimens decreased in order, as shown in Figure 12.

Since the analysis model was simplified, to precisely predict the average cross-section shrinkage of the specimens restrained by the steel plate and studs, a coefficient γ was introduced in Equation (18):

(18) $$\bar{\varepsilon }=\gamma \frac{\varepsilon_{\mathrm{sh}}}{\frac{E_{\mathrm{st}}}{E_{c}}•\frac{A_{3}}{A_{2}}+\frac{E_{t}}{\rho^{\prime }E_{c}+(1-\rho^{\prime })E_{t}}•\frac{A_{1}}{A_{2}}+1}$$

**Figure 13:**
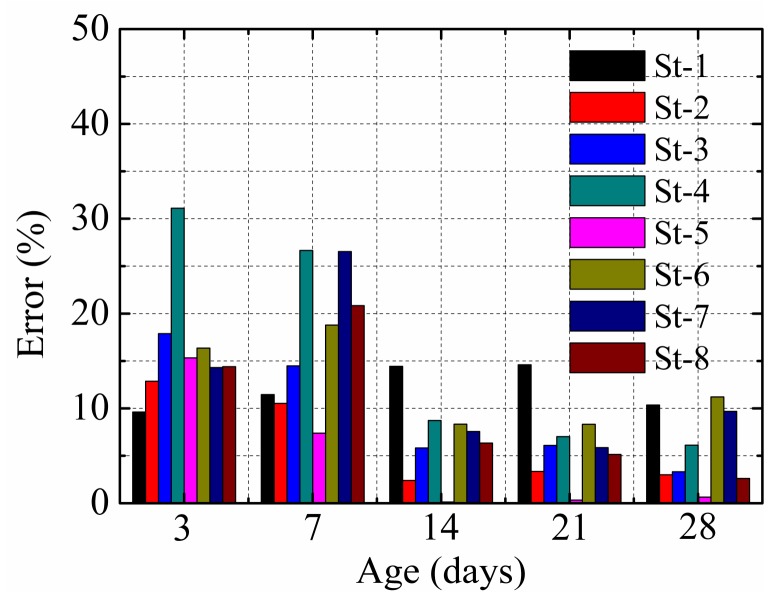
Calculation errors.

In accordance with the experimental results, in this study, γ = 1.1. Figure 13 presents the error of the predicted results at different ages. The average error was less than 20%. γ > 1 indicates that the analytical model overestimated the restraining effect of the stud.

**Figure 14:**
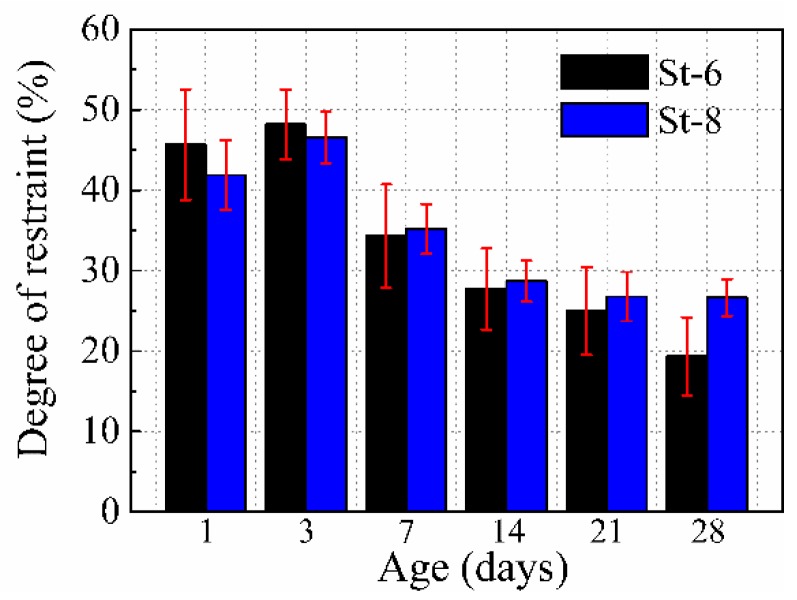
Average degrees of restraint at the 15, 75-c, 75-l, 75-r, 75-c, and 135 positions.

By adjusting the diameter and height of the stud, it was noted that the product of *E*_1_ and *A*_1_ for specimens St-6 and St-8 was almost the same. This would lead to the average cross section shrinkage of both the specimens to be similar, as shown in Figure 12. However, the shrinkage distribution in these two specimens was different. The restraining effect of the stud for an entire concrete cross-section can be expressed by the average degree of restraint for different measurement points on the same specimen. Figure 14 shows the comparison between the average degrees of the restraint of the specimens with the same product of *E*_1_ and *A*_1,_ but with different stud factors (St-6 and St-8). The average restraint degrees of these specimens were similar; however, the standard deviation for St-6 with a higher stud diameter (22 mm) and lower stud height (75 mm) was significantly higher than St-8 (16 and 115 mm, respectively). This indicates that with the same product of *E_1_* and *A_1_*, a lower diameter and higher height can provide a more uniform restraining effect on the entire concrete cross-section.

The strain obtained at positions 15, 75-c, and 135 are shown in Figure 11. When the height of the stud was increased, the strain obtained at 15, 75-c, and 135 decreased, and the strain gradient between positions 15 and 75-c, and that between positions 15 and 135 decreased as well. The restraining effect increased with the increase of diameter and number of studs. In addition, the strain obtained at the 75-c position in all specimens was lower than those at 75-l and 75-r, and the strain gradient between positions 15 and 75-c was lower than the strain gradient between positions 15 and 75-l, and that between positions 15 and 75-r. This indicated that the stud had a higher restraining effect depending on the position of the stud in the concrete. 

### 4.4. Cracking Potential

When the HPC shrinkage is restrained by a restraint, tensile stress takes place in the concrete. It will lead to cracking in concrete when the tensile strain of the concrete exceeds the ultimate tensile strain. Accordingly, the cracking potential of the concrete restrained by the restraint can be described using:

(19) $$P=\frac{\varepsilon_{\mathrm{sh}}-\varepsilon_{r}}{\varepsilon_{u}}$$

where *P* is the cracking potential of concrete and ε_u_ is the ultimate tensile strain of the concrete. 

The concrete cube compressive strength was used to calculate ε_u_ in this study [23], and the results are shown in Table 7 (the ultimate tensile strains of the concrete at ages that lay between the test ages for the concrete cube compressive strength were obtained using interpolation). Previous studies show that steel-plate-reinforced concrete elements are subject to biaxial stresses, even under uniaxial loading conditions [11]. Thus, the ε_u_ used in this study, which was obtained using the uniaxial test result, may present an error. However, since the length of the specimen was much larger than the width and height in this study, this error is acceptable.

**Figure 15:**
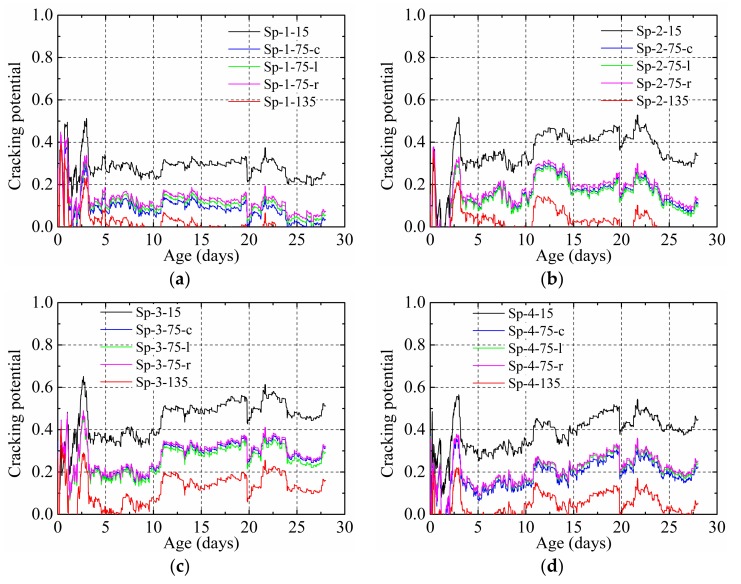
Cracking potential of the HPC restrained by steel plate: (**a**) Sp-1, (**b**) Sp-2, (**c**) Sp-3, and (**d**) Sp-4.

Figure 15 shows the cracking potential of the HPC restrained by the steel plate with different thicknesses and materials.

For the same measurement position in each specimen, higher cracking potentials were obtained for specimens with the same material when a higher steel plate thickness was used. At the same time, when the steel plate thicknesses were the same, a higher material elastic modulus of the steel plate led to a higher cracking potential of the HPC. These two phenomena imply that the cracking potential of the HPC was influenced by the restraining effect of the steel plate, a higher restraining effect, and a higher cracking potential.

For every specimen, the maximum and minimum cracking potential were obtained at positions nearest and farthest from the steel plate, respectively, because the effect of the steel plate in restraining the shrinkage in the HPC decreased with the increase of distance from the steel plate. Since the same distance to the steel plate implies the same restraining effect, the cracking potential obtained at 75-c, 75-l, and 75-r was very similar.

**Figure 16:**
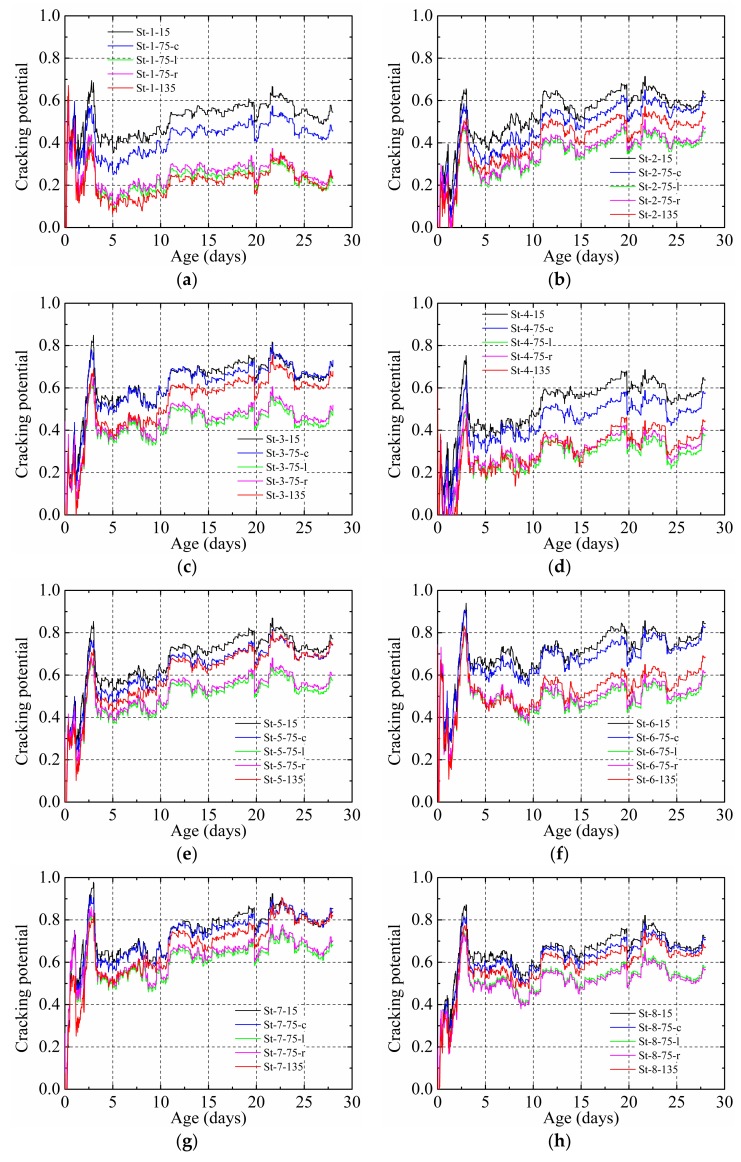
Cracking potential of the HPC restrained by steel plate and stud: (**a**) St-1, (**b**) St-2, (**c**) St-3, (**d**) St-4, (**e**) St-5, (**f**) St-6, (**g**) St-7, and (**h**) St-8.

Figure 16 shows the cracking potential of the HPC restrained by the steel plate and the stud with different stud factors. The cracking potential had the same change law as with the stud restraining effect. For each measurement position, a higher cracking potential was observed for a higher diameter, higher height, and when more studs were used. In addition, for each specimen, a higher cracking potential was obtained in the zone of influence of the stud.

**Figure 17:**
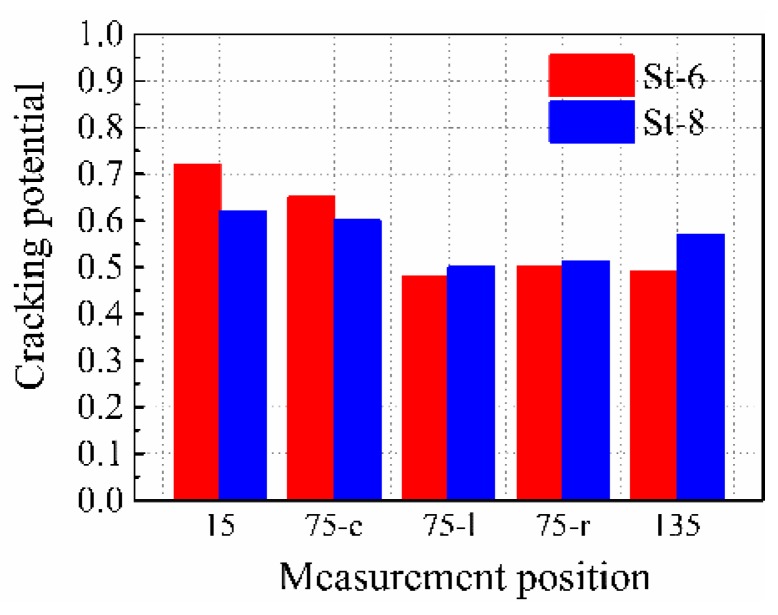
Average cracking potential of the specimens St-6 and St-8 throughout the age.

Figure 17 shows the average cracking potential of the specimens St-6 and St-8 throughout their age. It can be found that although the cracking potential for position 135 in St-8 increased with the increase in height of the stud, the maximum cracking potential of the specimens St-8 decreased to 0.62, which was 0.1 lower than that for specimens of St-6. Because the results demonstrate this, with the same product of *E_1_* and *A_1_*, using a lower diameter and higher height of studs to restrain the HPC more uniformly can decrease the maximum cracking potential of the HPC.

**Figure 18:**
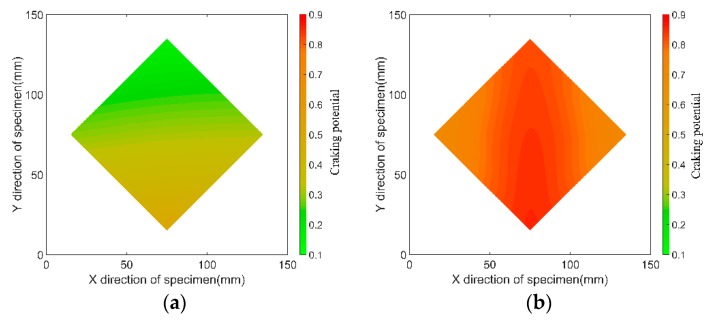
Distribution of the cracking potential of the specimens after 28 days: (**a**) Sp-3, and (**b**) St-7.

Figure 18 shows the distribution of cracking potential of the specimens Sp-3 and St-7 after 28 days. As shown in Figure 18a, when the HPC was only restrained by the steel plate, the cracking potential decreased with the increase of distance from concrete to steel plate. Thus, when the HPC was under a higher restraining effect (the tensile strain of concrete exceeded the ultimate tensile strain of concrete), the concrete would crack. Cracking is prone to happen from the surface of the steel plate.

The cracking potential in specimens restrained by the steel plate and the stud were higher than that for specimens restrained by only the steel plate. As shown in Figure 18b, when the stud was set into the specimens, the cracking potential in all cross-sections increased, especially in the area around the stud. The highest cracking potential occurred at the bottom of the stud because the highest restraining effect of the stud and steel plate occurred at this position. It is highly possible that the HPC would start cracking at the bottom of the stud, and it would develop along the boundary of the stud. In this scenario, the HPC will crack around the stud; this deduction conforms with the previous study phenomena and engineering practices [10].

## 5. Conclusions

This study has investigated the influence of a steel plate and studs on the shrinkage behavior and the cracking potential of HPC. According to the theoretical and experimental results, the main findings are concluded as follows.

- An analytical model of concrete restrained by studs and a steel plate was established on the basis of the unidirectional (length direction) analysis method. Theoretically, the strain of concrete restrained by the steel plate and stud decreased with the increase in the steel plate area and elasticity modulus of material, and the diameter, height, number, and elasticity modulus of the stud, when the size of the specimens and the free shrinkage of the concrete were constant. The influence of the area and material elasticity modulus of the steel plate, and height, diameter, and number of studs on the strain was experimentally analyzed. Recommended equations are given to predict the average cross section strain restrained by the steel plate only and steel plate and stud together.
- The lower strain and higher cracking potential occurred when a higher thickness and material elasticity modulus of the steel plate were used. The restraining effect of the steel plate on the concrete shrinkage decreased with an increase in the distance to the steel plate. An equation was given to calculate the strain distribution of HPC restrained by the steel plate. For specimens restrained using the steel plate, the highest cracking potential was obtained at the position where the steel plate was placed.
- The stud increased the cracking potential of the entire cross section of the HPC. The stud had a higher restraining effect on HPC where the stud was placed. With the same product of *E_1_* and *A_1_*, a lower diameter and higher height of the stud could restrain the HPC more uniformly and decrease the maximum cracking potential. When the HPC was restrained by the steel plate and the stud, the cracking of the HPC may have started from the bottom, and then developed along the boundary of the stud.

## Figures and Tables

**Table 1:** Proportions (kg/m^3^).

Mix	Water/Binder Ratio	Cement	Water	Fly Ash	Sand	Course Aggregate	Polycarboxylate Superplasticizer
HPC	0.21	385.0	116.0	165.0	704.0	1056.0	11.2

**Table 2:** Chemical compositions and physical properties of the cementitious materials.

Materials	Composition% (Mass)						Specific Surface (cm^2^/g)	Density (g/cm^3^)
	SiO_2_	CaO	Al_2_O_3_	Fe_2_O_3_	MgO	SO_3_		
Cement	21.47	65.77	5.47	4.28	1.44	0.52	3471	3.10
Fly Ash	49.47	4.45	20.67	14.32	1.17	1.40	4680	2.22

**Table 3:** List of experiments.

No.	Steel Plate Thickness (mm)	Stud Diameter (mm)	Stud Height (mm)	Stud Number (mm)	Restraint Material (Steel Plate/Stud)
P	–	–	–	–	–
Sp-1	4	–	–	–	Q345A/–
Sp-2	6	–	–	–	Q345A/–
Sp-3	10	–	–	–	Q345A/–
Sp-4	10	–	–	–	Q500-7/–
St-1	10	10	75	20	Q345A/ML15AL
St-2	10	10	135	20	Q345A/ML15AL
St-3	10	10	135	40	Q345A/ML15AL
St-4	10	16	75	20	Q345A/ML15AL
St-5	10	16	135	20	Q345A/ML15AL
St-6	10	22	75	20	Q345A/ML15AL
St-7	10	22	137	20	Q345A/ML15AL
St-8	10	16	115	20	Q345A/ML15AL

**Table 4:** Properties of the steel plate and studs.

Type	Material	Elastic Modulus (×10^4^ MPa)	Yield Strength (MPa)	Ultimate Strength (MPa)
Steel plate	Q345A	20.6	390.0	555.0
	Q500-7	15.4	320.0	500.0
Stud	ML15AL	19.0	419.0	520.3

**Table 5:** Cube compressive strength and elastic modulus of HPC.

Basic Properties	Age (Days)				
	3	7	14	21	28
Cube compressive strength (MPa)	67.1	77.9	78.5	84	85.8
Elastic modulus (×10^4^ MPa)	5.52	5.68	6.03	6.22	6.42

**Table 6:** Comparing of test value and calculated value (με).

Specimens	7 Days			14 Days			21 Days			28 Days		
	Test	Calculated	Error	Test	Calculated	Error	Test	Calculated	Error	Test	Calculated	Error
Sp-1	312	310	0.6%	405	410	1.2%	440	461	4.8%	466	494	6.0%
Sp-2	300	306	2.0%	388	384	1.0%	422	440	4.3%	447	484	8.3%
Sp-3	276	297	7.6%	358	372	3.9%	389	418	7.5%	412	441	7.0%
Sp-4	289	307	6.2%	376	399	6.1%	408	430	5.4%	433	457	5.5%

**Table 7:** Ultimate tensile strain of concrete.

ε_u_	Age (Days)				
	3	7	14	21	28
Ultimate tensile strain (με)	182	196	197	203	205
